# Medical Electricity: Its Application to Medicine and Surgery

**Published:** 1887-06

**Authors:** 


MEDICAL ELECTRICITY: A PRACTICAL TREATISE ON THE APPLICATION OF ELECTRICITY TO MEDICINE AND SURGERY. *By* ROBERTS BARTHOLOW, A.M., M.D., LL.D. *Third Edition, Enlarged and Improved. Illustrated, pp.* XX *and 304. Philadelphia*: LEA BROTHERS & COMPANY. *Chicago:* A. C. MCCLURG & COMPANY, 1887.

Those who are already familliar with this book need only to know that the additions embodied in this edition are highly practical and occur chiefly in the therepeutical sections, bringing the work fully up to the present, to the standard of our knowledge of the subject. Quite a full account is given of appliances for electrical illumination, and galvano-cautery, and galvano-faradization and electric baths receive appropriate consideration. To those who are not already familiar with the book in its former editions, it may be said, that for medicinal purposes it is amply full, and is characterized throughout by practical good sense and directness.

